# A possible important regulatory role of estrogen in obstructive sleep apnea hypoventilation syndrome

**DOI:** 10.3389/fmed.2025.1369393

**Published:** 2025-03-03

**Authors:** Pinyi Zhou, Hongmei Li, Hongyan Li, Yan Chen, Yunhui Lv

**Affiliations:** ^1^Department of Sleep Medicine, The Affiliated Hospital of Kunming University of Science and Technology, The First People's Hospital of Yunnan Province, Kunming, China; ^2^Department of Neurology, Yan'an Hospital Affiliated to Kunming Medical University, Kunming, China

**Keywords:** sleep apnea, obstructive, CIH, estrogen, ERα, HIF-1α

## Abstract

Obstructive sleep apnea-hypoventilation syndrome (OSAHS) is a prevalent clinical sleep breathing disorder that affects both pediatric and adult populations. If left untreated, OSAHS can induce or aggravate systemic dysfunction across multiple organ systems, with a particularly pronounced impact on cardiovascular health, thereby posing a substantial threat to overall human well-being. Notably, there exists a significant sex disparity in the prevalence and severity of OSAHS, with a higher incidence and greater severity observed in males. However, this disparity tends to diminish post-menopause. Research indicates that sex differences in OSAHS are associated with gonadal function, wherein estrogen exerts a protective effect by modulating pharyngeal muscle tone and mitigating oxidative stress. This regulatory role of estrogen partially reduces the incidence of OSAHS and attenuates its pathological impact. Conversely, OSAHS may adversely affect gonadal function, resulting in decreased estrogen levels, which can exacerbate the condition. This review examines the beneficial role of estrogen in the progression of OSAHS and explores the potential impact of OSAHS on estrogen levels.

## 1 Introduction

OSAHS is primarily characterized by the recurrent partial or complete obstruction of the upper airway during sleep, leading to chronic intermittent hypoxemia and associated pathophysiological consequences, including disrupted sleep architecture, prolonged sympathetic activation, and carbon dioxide retention (1). Epidemiological data suggest that the prevalence of OSAHS is on the rise annually, with an estimated 425 million adults aged 30 to 69 years affected by moderate to severe forms of the condition worldwide (2). Furthermore, the prevalence is notably higher in males compared to premenopausal females, with a male-to-female ratio of approximately 2:1 (3, 4). In females, the incidence of OSAHS increases with age, particularly following menopause (3, 5, 6), exhibiting minimal differences when compared to males (1, 7). For instance, the HypnoLaus cohort study conducted by Heinzer et al. (8) (*n* = 2,121) revealed a significantly greater prevalence of OSAHS in males compared to premenopausal females (83.8% vs. 35.1%). However, this prevalence gap was considerably reduced when comparing males to postmenopausal females (83.8% vs. 71.6%). Additionally, the prevalence of OSAHS was markedly higher in postmenopausal females not undergoing hormone replacement therapy than in both premenopausal females and postmenopausal females receiving hormone replacement therapy, particularly when controlling for confounding variables such as body mass index (BMI) and neck circumference (5, 6). Alarmingly, over 90% of perimenopausal and postmenopausal females with OSAHS remain undiagnosed (8). In comparison to premenopausal females, postmenopausal females with OSAHS exhibit more severe symptoms and elevated apnea-hypopnea index (AHI) levels (9, 10), with serum estradiol (E2) levels demonstrating a significant negative correlation with both AHI and arousal index (11).

Research has indicated that hormone replacement therapy is associated with a decreased prevalence of sleep apnea in postmenopausal women, even after controlling for confounding variables such as age, body mass index (BMI), and neck circumference (12). Notably, estrogen, which is crucial for the physiological response and adaptation to hypoxic conditions (13), significantly influences the gender-specific effects of obstructive sleep apnea-hypopnea syndrome (OSAHS) on cardiovascular outcomes. It has been shown to lower the incidence of cardiovascular diseases associated with OSAHS, as well as related morbidity and mortality (4, 14), and to mitigate the risk of depression (8, 11). Estrogen’s protective role in OSAHS operates through various mechanisms, and a comprehensive investigation of these mechanisms could provide a theoretical foundation for identifying new therapeutic targets for OSAHS. Such insights could enhance clinicians’ understanding of sleep-related disorders and their complications in postmenopausal women, thereby reducing the likelihood of missed diagnoses and delays in clinical intervention.

**Figure 1:**
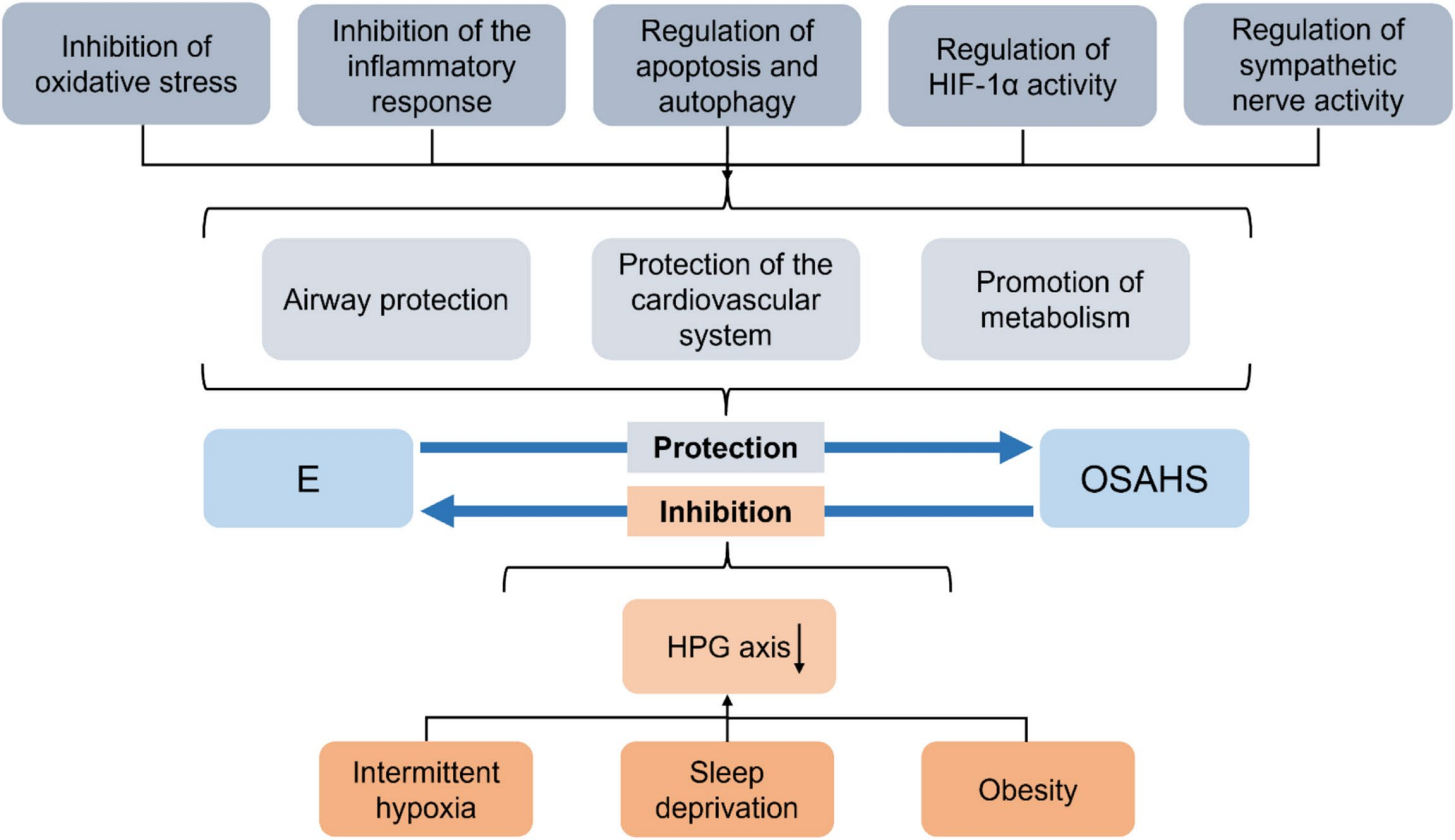
Summary of the interaction between OSAHS and estrogen. E, estrogen; OSAHS, obstructive sleep apnea-hypopnea syndrome; HPG, hypothalamic–pituitary-gonadal.

In addition, a chronic systemic inflammatory response in OSAHS adversely affects multiple organs throughout the body. Despite the protective effect of estrogen on OSAHS, it also suffers from the pathologic damage of OSAHS (Figure 1). Significantly, sleep deprivation disrupts the circadian rhythm of estrogen secretion and causes a variety of endocrine disorders, leading to a decline in estrogen levels (15). In theory, this would further exacerbate the condition of OSAHS. As there are few studies on this, we discuss the adverse effects of estrogen in terms of the common pathomechanisms of OSAHS, aiming to provide ideas for future research.

## 2 Important protective role of estrogen in OSAHS

**Figure 2:**
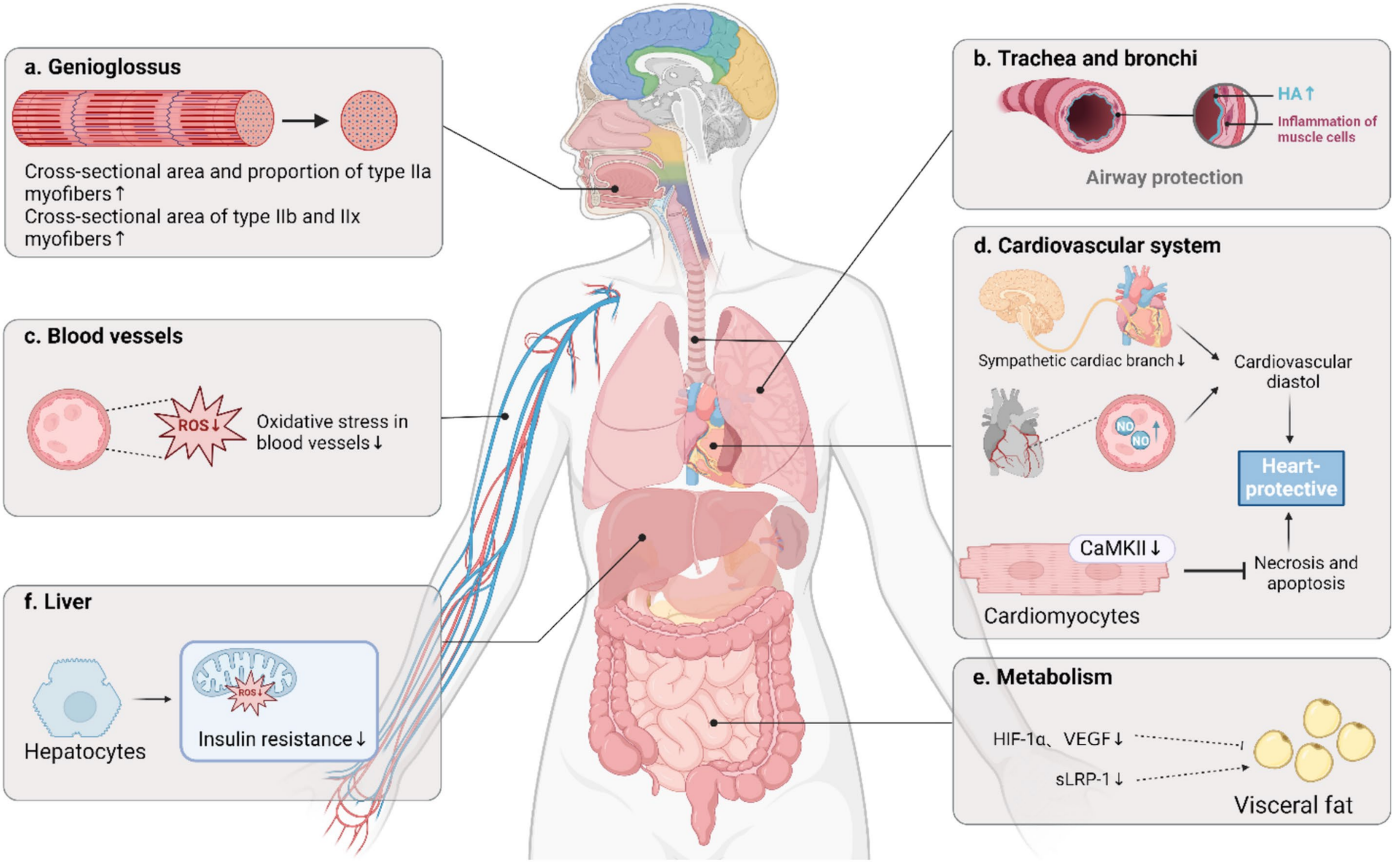
Important protective role of estrogen in OSAHS. OSAHS: Obstructive sleep apnea-hypopnea syndrome; HA, hyaluronic acid; ROS, reactive oxygen species; CaMKII, calcium/calmodulin-dependent protein kinase II; HIF-1α, hypoxia-inducible factor-1α; VEGF, vascular endothelial growth factor; sLRP-1, soluble low-density lipoprotein receptor-related protein-1. Created in BioRender.

The most biologically active form of estrogen is estradiol (E2), whose genomic effects are facilitated by two receptor subtypes, estrogen receptor alpha (ERα) and estrogen receptor beta (ERβ), both of which are classified as nuclear receptors. The distribution and expression levels of ERα and ERβ differ across various tissues, which underlies the diverse biological effects of estrogen (16). It has been established that E2 exerts its protective effects on OSAHS primarily through ERα and Erβ (Figure 2).

### 2.1 Airway protection

Sex hormones play a protective role in maintaining the patency of the upper airway in premenopausal females. The condition of the upper airway is influenced by the interplay between the positive intraluminal pressure that keeps the airway open and the opposing surface tension that tends to close it (17). Research indicates that upper airway collapsibility may be more pronounced in males than females, as evidenced by measurements of critical closure pressure, defined as the pressure at which airflow ceases due to airway collapse (18). The pharyngeal muscle, a key skeletal muscle responsible for dilating the upper airway, significantly impacts airway patency through its functional status. This muscle is particularly important in the pathogenesis of OSAHS, especially regarding its pathomorphological characteristics (19). The genioglossus muscle is crucial for maintaining an open airway to facilitate adequate respiration among the pharyngeal muscles (17, 20). Furthermore, exposure to chronic intermittent hypoxia (CIH) enhances the body’s ventilatory response to hypoxia, leading to prolonged respiratory motor output, whether sublingual or diaphragmatic. This prolonged output can increase the susceptibility of skeletal muscles to fatigue and atrophy, ultimately impairing muscle function and potentially exacerbating sleep apnea (21).

The functional characteristics of mammalian skeletal muscle are influenced by the various types of muscle fibers present, with the rate of force production, fatigue resistance, and energy metabolism being contingent upon the composition of these fibers, which include both slow and fast types (21, 22). Slow muscle fibers, classified as type I, and fast muscle fibers, categorized as type II (encompassing types IIa, IIb, and IIx), exhibit distinct physiological properties. In healthy individuals, approximately 90% of the pharyngeal muscles consist of fatigue-resistant fibers (types I and IIa), while only 10% comprise fatigue-sensitive fibers (type IIb). The predominance of fatigue-resistant fibers in human pharyngeal muscle can be attributed to the high mitochondrial content of type I fibers, which utilize fatty acids oxidatively to generate acetyl-coenzyme A, and the enhanced oxidative capacity of type IIa fibers (23). Conversely, individuals with OSAHS demonstrate a reduced ratio of type I to type IIb fibers, alongside an increased proportion of type IIa fibers (23). Notably, the ratio of type I fibers is positively correlated with the lowest oxygen saturation levels (24), contributing to pharyngeal muscle fatigue and an increased vulnerability to pharyngeal collapse during sleep (21, 23, 25). Research indicates that CIH leads to a diminished contractile function of the genioglossus muscle, a condition that is further aggravated by menopause in human females (26) or ovariectomy (OVX) in rats (27, 28). Estrogen has been shown to enhance myoelectric activity and tonic muscle tone in the genioglossus (26–28), counteracting the reduction in cross-sectional area and proportion of type IIa fibers associated with estrogen deficiency and hypoxia, while also increasing the cross-sectional area of type IIb and IIx fibers (27). These findings suggest that estrogen plays a significant role in the positive regulation of genioglossus muscle function.

ERα is extensively present in mouse skeletal muscle, and it is posited that estrogen may enhance muscle endurance by modulating muscle fiber composition via the ERα signaling pathway (24). Research conducted by Cabelka et al. (29) demonstrated that mice with a skeletal muscle-specific knockout of ERα exhibited increased susceptibility to fatigue compared to control mice, and they showed impaired recovery of strength, indicating the critical role of ERα in mitigating skeletal muscle fatigue and facilitating recovery. Additionally, a significant concentration of ERα is found in the genioglossus muscle, where E2 influences its contractility primarily through the ERα pathway, while also promoting the expression of ERα mRNA and protein, with no observed effect on ERβ (30). Chen et al. (24) reported a reduction in the expression levels of ERα and type I muscle fibers in the palatopharyngeal tissues of patients with OSAHS, revealing a significant positive correlation between the two. Most studies indicate that OSAHS is linked to an increase in type II muscle fibers and a decrease in type I fibers. As the severity of OSAHS progresses, there is a gradual decline in the proportion of type I fibers, accompanied by a corresponding increase in type II fibers (21). However, Chen et al. (24) specifically noted an increase in type IIx muscle fibers in the palatopharyngeal tissues of OSAHS patients, rather than an increase in type II fibers. Furthermore, animal studies have shown that ERα expression and a decrease in type I muscle fibers were observed in the sternohyoid muscle of OVX rats, with no significant alterations in type IIb muscle fiber expression (24). The underlying changes in muscle fiber types associated with OSAHS remain unclear and may be influenced by various factors, including age, sex, and the specific biopsy site. The direct contribution of these fiber type changes to OSAHS is still uncertain. Current evidence suggests a beneficial effect of estrogen on skeletal muscle contractility; however, further research is warranted to confirm this effect and to investigate the mechanisms through which estrogen interacts with different receptors.

A recent investigation revealed diminished plasma concentrations of hyaluronic acid (HA) and markedly elevated levels of hyaluronidases (HYAL) in individuals diagnosed with OSAHS (31). HA, a glycosaminoglycan, serves as a crucial component of the extracellular matrix within tracheal and bronchial mucosa and endothelial cells (32, 33). High-molecular weight HA (HMW-HA) functions as a vital anti-inflammatory and antioxidant agent, whereas low molecular weight HA is associated with promoting inflammatory processes (34). The metabolism of HA is primarily facilitated by HYAL, particularly HYAL1 and HYAL2, which are present in various tissues, including the lungs. This metabolic process generates low molecular weight fragments that can exacerbate inflammatory responses, thereby intensifying inflammatory damage (35). Alterations in HA synthesis and metabolism have been identified in airway diseases characterized by chronic inflammation and oxidative stress, such as bronchial asthma and chronic obstructive pulmonary disease (COPD). These alterations increase lung inflammation and remodeling, reducing lung compliance and airway obstruction (33, 35, 36). Klagas et al. (33) reported that HA levels were significantly lower in primary human airway smooth muscle cells derived from patients with asthma and COPD. This reduction was correlated with a notable decrease in HA synthase-1 and -2 expression, alongside a significant increase in HYAL1, indicating a suppression of HA synthesis and an enhancement of its catabolism. Furthermore, Klagas et al. observed a decline in CD44 receptor expression, which hindered the clearance of HA degradation products, thereby perpetuating inflammation (33). Meszaros et al. (31) found a significant negative correlation between plasma levels of HMW-HA and the apnea-hypopnea index (AHI) in patients with OSAHS, while HYAL-1 exhibited a significant positive correlation with both AHI and the oxygen desaturation index (ODI). This suggests that chronic hypoxia is linked to elevated plasma concentrations of HYAL-1 and accelerated degradation of HMW-HA (31). Additionally, one study indicated that estrogen treatment resulted in increased skin HA levels in mice, an effect that could be inhibited by estrogen receptor antagonists, implying that estrogen elevates HA levels through the activation of its specific receptors (37). However, more studies need to address this topic, necessitating further investigation into the alterations in lung HA levels among OSAHS patients. It is also imperative to consider the effects of sleep deprivation and to evaluate changes in these indices following treatments such as continuous positive airway pressure (CPAP) and estrogen therapy. Such research will enhance our understanding of HA’s role in airway inflammation in OSAHS and provide additional evidence regarding the airway protective effects of estrogen.

### 2.2 Promotion of metabolism

In postmenopausal women and the OVX rat model, a reduction in estrogen levels has been linked to the onset of central obesity, dyslipidemia, insulin resistance, and an elevated risk of developing non-alcoholic fatty liver disease, type 2 diabetes, and cardiovascular disease (10, 38, 39). Following the decline in ovarian estrogen production in postmenopausal women, estrone becomes the predominant form of estrogen, primarily synthesized through the peripheral aromatization of androstenedione in muscle and adipose tissues. This process often increases body mass and visceral fat accumulation during the postmenopausal phase (40–42). A metabolic syndrome characterized by visceral obesity is associated with the onset and progression of OSAHS (1, 8). Research indicates that CIH further contributes to an increase in body mass (43) and a reduction in rectal temperature (44) in OVX rats, implying that OSAHS may exacerbate the hypo-metabolic condition induced by decreased estrogen levels.

The regulation of metabolism by estrogen is predominantly mediated through ERα. Administration of E2 has been shown to enhance mitochondrial function in the liver while simultaneously decreasing the production of reactive oxygen species (ROS) and mitigating insulin resistance in rat models (45). Furthermore, E2 treatment reduces the expression of hypoxia-inducible factor-1α (HIF-1α) and vascular endothelial growth factor in the periaortic and intra-abdominal adipose tissue of OVX rats. This reduction contributes to decreased visceral fat accumulation and improved insulin sensitivity, as evidenced by restored blood glucose and serum leptin levels, thereby providing a comprehensive improvement in metabolic syndrome (39). Additionally, multiple studies have substantiated the beneficial effects of E2 on metabolic and visceral obesity and its protective role in OSAHS (43, 44). However, Boukari et al. (46) conducted a study involving OVX rats exposed to CIH and treated with selective ERα receptor agonists, which revealed no significant alterations in body mass. This lack of change may be attributed to the brief duration of hypoxic exposure.

Recent research has indicated that soluble low-density lipoprotein receptor-related protein-1 (sLRP-1) levels are significantly diminished in patients diagnosed with OSAHS. This reduction has been correlated with severe nocturnal hypoxia and disrupted lipid metabolism *in vivo* (47). sLRP-1 plays a crucial role in anti-inflammatory and metabolic processes within the body, and its low concentrations are linked to metabolic irregularities associated with OSAHS, implying its function as a protective protein. It has been suggested that OSAHS may elevate LRP1 expression through the action of HIF-1α, which could result in the accumulation of low-density lipoprotein cholesterol esters in cardiomyocytes (48) and vascular smooth muscle cells (49). It is plausible that other inhibitory mechanisms may counteract this stimulatory effect, leading to the observed decline in sLRP-1 concentrations in OSAHS. The inhibitory influence of estrogen on HIF-1α is discussed further, and we postulate that estrogen may suppress sLRP1 expression. This hypothesis was substantiated by an animal study involving tilapia, which demonstrated a significant reduction in LRP1 levels following treatment with a high dose (50 mg/kg) of E2 accompanied by hepatic lipid accumulation (50). The conclusion must be considered with caution due to species differences, which suggests that we need to pay attention to the dual effects of estrogen on OSAHS metabolism. The mechanism of interaction between estrogen and adipose function and metabolic disorders needs to be clarified by further studies.

Conversely, postmenopausal women who received estrogen therapy exhibited greater physical activity and engaged in higher levels of moderate to vigorous exercise compared to their untreated counterparts (51). This indicates that estrogen is crucial in modulating physical activity levels following menopause. In a study conducted by Cabelka et al. (29), OVX rats were administered estrogen, progesterone, and a combination of both hormone treatments, with subsequent comparisons regarding changes in activity levels before and after treatment. The findings revealed that the activity levels of rats receiving estrogen and the combined hormone treatment were significantly elevated compared to the control group, with the increase particularly pronounced in the latter. These results suggest that estrogen is the primary ovarian hormone influencing physical activity, while progesterone may enhance the effects of estrogen. Estrogen can potentially elevate metabolism to a certain degree by influencing physical activity levels.

### 2.3 Protection of the cardiovascular system

OSAHS has been associated with numerous adverse effects on the cardiovascular system, potentially inducing or exacerbating pre-existing conditions (1). This phenomenon is particularly pronounced in postmenopausal women, who demonstrate an elevated risk for hypertension, arrhythmias, and heart failure (52, 53).

Research conducted by Lan et al. (14) indicates that the surgical removal of ovaries in rats leads to hypoxia-induced oxidative stress and damage to the vascular endothelium. This damage is characterized by disorganization, hypertrophy, and proliferation of vascular smooth muscle cells, as well as the destruction of endothelial cells and thickening of the middle layer of the endothelium, all of which contribute to an increased risk of atherosclerosis. Estrogen has been shown to mitigate vascular oxidative stress in OVX rats subjected to intermittent hypoxia (3). Furthermore, treatment with E2 may offer protective benefits against vascular complications in female patients with OSAHS (46). Additionally, ERαis highly expressed in the carotid body and central structures that regulate sympathetic nerve activity and vascular function (54). E2 has been found to decrease cardiac output, heart rate, and arterial pressure during CIH exposure by activating ERα, which in turn inhibits the activation of the cardiac branch of the sympathetic nervous system and enhances nitric oxide-mediated vasodilatory responses (3).

Heart failure represents a significant comorbidity associated with OSAHS. Research indicates that female patients with OSAHS exhibit a higher propensity for developing heart failure with preserved ejection fraction compared to those with heart failure characterized by reduced ejection fraction (53, 55). Furthermore, echocardiographic parameters have been found to correlate significantly with the severity of OSAHS. Notably, diastolic left ventricular filling is more frequently compromised in female patients than in their male counterparts, with this impairment showing a significant correlation to minimum oxygen saturation levels and the duration of oxygen saturation below 90%. Additionally, ventricular diastolic sarcoplasmic reticulum calcium leakage has been closely linked to ventricular systolic dysfunction and arrhythmias. Lebek et al. (56) reported an increase in calcium/calmodulin-dependent protein kinase II (CaMKII)-induced sarcoplasmic reticulum calcium leakage among patients with OSAHS, a phenomenon potentially associated with elevated production of ROS resulting from the condition. E2 has been shown to confer cardioprotective effects by mitigating oxidative stress, reducing ROS production, inhibiting CaMKII expression, and decreasing cardiomyocyte necrosis and apoptosis (57). Consequently, diminished levels of estrogen render postmenopausal female patients with OSAHS more vulnerable to severe cardiovascular complications, while E2 appears to alleviate the cardiovascular damage associated with OSAHS.

## 3 Important protective mechanisms of estrogen in OSAHS

**Figure 3:**
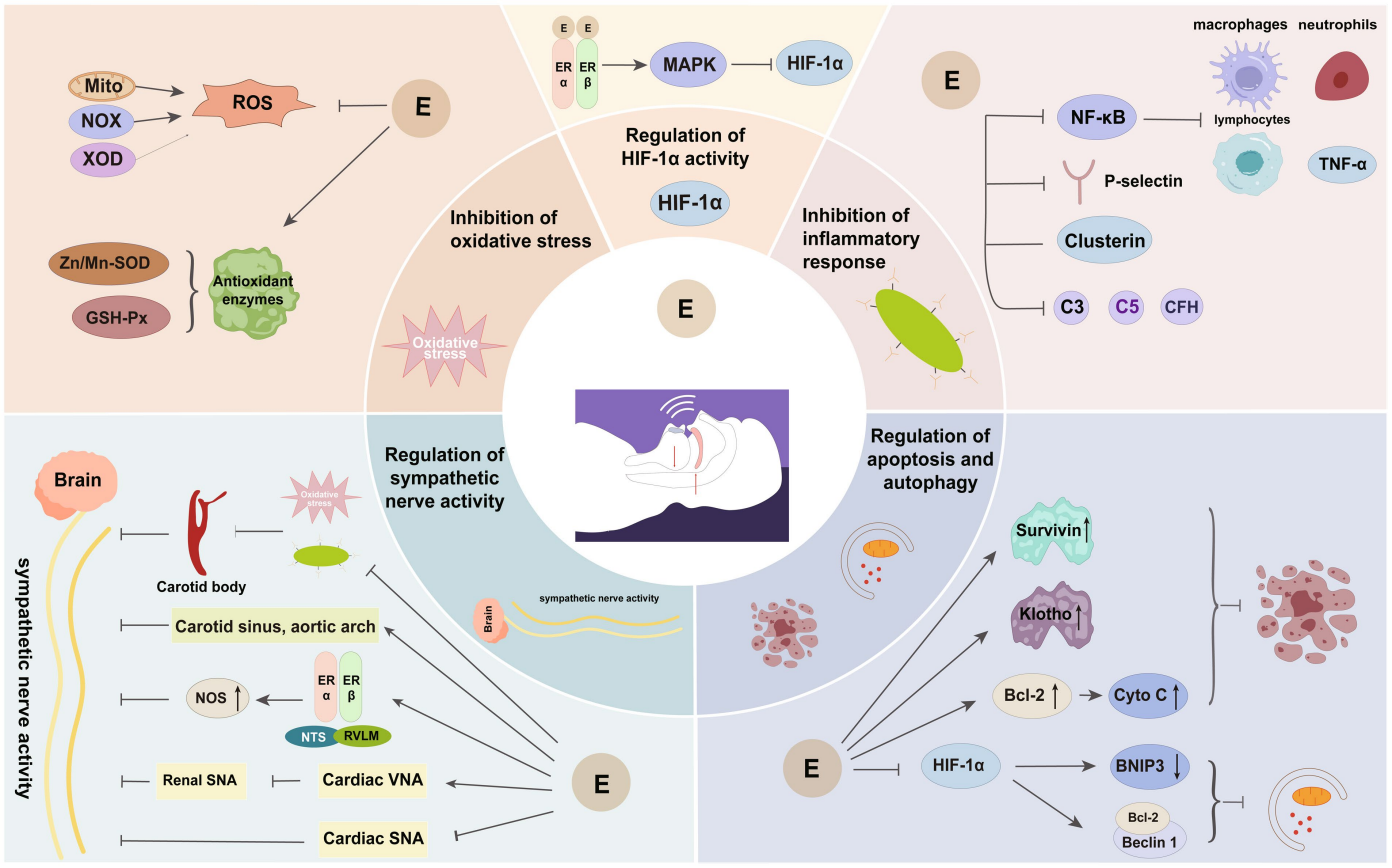
Important protective mechanisms of estrogen in OSAHS. E, estrogen; Mito, mitochondria; NOX, reduced nicotinamide adenine dinucleotide phosphate oxidase; XOD, xanthine oxidase; ROS, reactive oxygen species; Zn/Mn-SOD, zinc-manganese superoxide dismutase; GSH-Px, glutathione peroxidase; MAPK, mitogen-activated protein kinase; HIF-1α, hypoxia-inducible factor-1α; NF-κB, nuclear factor kappa B; CFH, complement factor H; Bcl-2, B cell lymphoma-2; Cyto C, cytochrome c; NTS, nucleus tractus solitarius; RVLM, rostral ventrolateral medulla; NOS, nitric oxide synthase; VNA, vagus nerve activity; SNA, sympathetic nerve activity.

The presence of considerable oxidative stress, inflammatory responses, and sympathetic activation in OSAHS has the potential to harm various tissues and organs within the body. Estrogen may play a protective role by mitigating these pathological response mechanisms (Figure 3).

### 3.1 Inhibition of oxidative stress

An imbalance between pro-oxidants and antioxidants can result in tissue and organ damage, known as “oxidative stress.” This imbalance may arise from excessive production of ROS or deficiencies in the antioxidant defence mechanisms (14). The primary sources of ROS include mitochondria, NADPH oxidase, and xanthine oxidase (58, 59). In patients with OSAHS, recurrent nocturnal hypoxemia leads to significant ROS production and diminished antioxidant capacity within the organism. The accumulation of excessive ROS can induce cellular damage (60–62), impairing tissues and organs’ normal functioning.

E2, recognized as the most biologically active form of estrogen, has been shown to exhibit antioxidant properties. It promotes the synthesis of antioxidant enzymes by binding to specific receptors that enhance the expression of nuclear genes related to antioxidant enzymes within mitochondria, thereby reducing intracellular levels of ROS (44, 59, 63). A substantial body of research indicates that CIH exposure induces oxidative stress across various tissues, including lung tissue, the cerebral cortex, the brainstem, adrenal glands, vascular endothelium, and other organs in OVX rats. Furthermore, E2 supplementation has been found to mitigate the elevated oxidative stress levels induced by CIH significantly and to enhance the activity of antioxidant enzymes, such as zinc/manganese superoxide dismutase and glutathione peroxidase, which protect against oxidative stress-related damage (14, 44, 64, 65). In a study conducted by Ribon-Demars et al. (61), E2 treatment was observed to increase the activity of antioxidant enzymes in the tissues of OVX rats while concurrently exacerbating oxidative stress levels. This observation suggests that E2 may stimulate mitochondrial ROS production, potentially influenced by the concentration of supplemental estrogen or the distribution of receptors; however, the overall effect on oxidative stress appears to be inhibitory. Irwin et al. (64) further clarified that only ERβ, and not ERα, affected the expression of genes encoded by mitochondrial DNA in brain tissue, with ERα activation serving as the primary mechanism for initiating the scavenging of lipid peroxides in brain mitochondria. Consequently, there are notable differences in the distribution and function of various estrogen receptor subtypes across different tissues, with ERβ playing a pivotal role in mitigating oxidative stress.

### 3.2 Inhibition of the inflammatory response

In the context of OSAHS, CIH activates the nuclear factor-κB (NF-κB) and HIF-1αsignaling pathways, with NF-κB serving as a pivotal transcription factor in the inflammatory response (62). NF-κB can also induce muscle atrophy, leading to the loss of slow-twitch muscle fibers and affecting muscle tone (66). Estrogen has been shown to inhibit NF-κB activity by activating its receptor (67). Research indicates a pronounced inflammatory response in the lung tissues of female rats subjected to hypoxia, characterized by a significant increase in the nuclear levels of the NF-κB p65 subunit protein, which was further exacerbated following ovariectomy (13). Additionally, a notable inflammatory response was detected in the bronchoalveolar lavage fluid of ovariectomized rats exposed to CIH, evidenced by elevated levels of total proteins, inflammatory cells (including macrophages, neutrophils, and lymphocytes), tumor necrosis factor-alpha (TNF-*α*), and lipid peroxides (13). Conversely, a study conducted by Torres et al. (43) found that while intermittent hypoxia intensified the inflammatory response (as indicated by the overexpression of IL-6 and IL-8 genes) in the cardiac and cerebral tissues of rats, it did not further elevate inflammatory markers in ovariectomized rats, aligning with the observations of Ribon-Demars et al. (61). This phenomenon may be attributed to estrogen’s role in stabilizing HIF-1α activity, which activates NF-κB to facilitate inflammatory responses (62). Under hypoxic conditions, estrogen enhances the stability of HIF-1αand promotes NF-κB activation, thereby increasing the expression of IL-6 and IL-8. In contrast, a reduction in estrogen levels leads to diminished HIF-1α activity in ovariectomized rats, resulting in decreased NF-κB activity and the expression of its target genes, IL-6 and IL-8 (43). However, numerous studies have demonstrated that E2 can inhibit HIF-1α expression via ERα (17, 28, 68) or ERβ (69), presenting contradictory evidence that suggests the existence of alternative pathways through which estrogen may influence HIF-1α. Notably, Torres did not further investigate this inflammatory response mechanism through estrogen replacement therapy, whereas Huang et al. (13) reported a significant reduction in the inflammatory response following the administration of a low dose of E2 to ovariectomized rats, indicating a more reliable outcome and suggesting that estrogen may mitigate hypoxia-induced inflammatory responses in the lung.

Numerous inflammatory diseases are characterized by increased recruitment of immune cells to vascular sites. P-selectin, an inflammatory adhesion molecule, plays a crucial role in this mechanism, particularly atherogenesis (70). Research conducted by Horváth et al. (71) demonstrated that plasma levels of P-selectin were significantly elevated in patients suffering from severe OSAHS, although these levels did not correlate with the arousal index. This finding indicates that P-selectin is implicated in the inflammatory response associated with OSAHS, which is influenced by nocturnal hypoxia. Furthermore, estrogen has been shown to modulate P-selectin levels; for instance, P-selectin levels decrease in response to elevated E2 during the menstrual cycle in females, and similarly, levels in males decline following intramuscular E2 administration (72). This observation provides additional evidence supporting the anti-inflammatory properties of estrogen.

Additionally, clusterin, a heterodimeric protein known for its anti-apoptotic and anti-inflammatory functions (73), is involved in the inflammatory processes related to OSAHS. Elevated clusterin levels in OSAHS patients have been linked to nocturnal hypoxic stimulation and have shown a positive correlation with the severity of the disease (74). This suggests clusterin may be a protective mechanism against OSAHS and could partially inhibit NF-κB activity (75). However, a study indicated that estrogen downregulates clusterin gene expression in the rat endometrium (76), which appears to contradict the anti-inflammatory effects of estrogen. It is essential to consider the tissue-specific variability in estrogen action, which may be influenced by the distribution of its receptor subtypes (77). Further research is necessary to validate these findings.

The overexpression of complement components C3, C5, and C9 has been observed in patients with OSAHS (78), indicating an activation of the complement system (78, 79). This system plays a crucial role in the hazard perception cascade response and is integral to humoral innate immunity, encompassing functions such as antimicrobial defense, immunomodulation, clearance of immune complexes, and apoptosis (80, 81). Research conducted by Horvath et al. (79) identified a significant association between elevated levels of C3a and CIH in OSAHS patients; however, no notable alterations were detected in the levels of C5a or SC5b-9. C3a is generated through the cleavage of C3 by the C3 converting enzyme, while the binding of C5b-9, known as the membrane attack complex (MAC), to S-protein results in the formation of the inactive stabilized form SC5b-9 (80). The activation of the complement system may be linked to a decrease in the expression of complement component 4-binding protein alpha in OSAHS (78), as this protein serves as an inhibitor of both the classical and clusterin pathways and obstructs the formation of C3b and C4b2b. The stable levels of plasma C5a and SC5b-9 suggest a potential attenuation of the complement cascade or the existence of a protective feedback mechanism in OSAHS (79). Complement factor H (CFH) is a crucial regulator of the alternative pathway within the complement system, inhibiting the activity of the C5 converting enzyme, reducing C5 cleavage, and exerting anti-inflammatory effects (81). Nevertheless, no significant changes in CFH levels were noted in OSAHS patients, which complicates understanding the observed attenuation of the complement cascade. Additionally, clusterin, which inhibits MAC formation and thereby mitigates cytolysis and the inflammatory response (81), may account for the unchanged levels of SC5b-9. The proposition of a sluggish complement cascade necessitates further investigation. Lastly, the impact of estrogen on the complement system has been evaluated, albeit with limited studies available. One investigation indicated that estrogen enhances C3 secretion in rat uterine epithelium, a phenomenon not replicated in the liver (82), suggesting that the effects of estrogen may be tissue-specific and cannot be generalized as universally activating the complement system. Further research is warranted to elucidate this relationship.

The inflammatory response associated with OSAHS is intricate, and there exists a diversity of perspectives regarding the influence of estrogen. Comprehensive research is required to investigate this phenomenon across various tissues and organs, allowing for adequate consideration of environmental exposures. Such investigations are essential to elucidate whether the inflammatory response is tissue-specific or if it involves more profound molecular mechanisms. Furthermore, clinical studies should focus on monitoring changes in pertinent indices following OSAHS treatment while also accounting for the impacts of obesity, sleep deprivation, and other confounding variables.

### 3.3 Regulation of apoptosis and autophagy

CIH is a well-established trigger for apoptosis, with numerous studies indicating that hypoxia prompts pharyngeal myocytes in rat models to generate ROS excessively. This overproduction subsequently influences the expression of genes associated with apoptosis, ultimately resulting in organ dysfunction (20, 21, 62). Survivin (83) and Klotho (84) are pivotal anti-apoptotic proteins in inflammation and immune regulation. In individuals diagnosed with OSAHS, hypoxia has been shown to inhibit these two proteins, with their regulation being influenced by estrogen; however, there is a notable absence of data regarding their status following CPAP treatment. Kunos et al. (85) were the first to document the impact of OSAHS on Survivin levels, revealing a significant reduction in plasma Survivin among OSAHS patients, which correlated with nocturnal hypoxia and elevated C-reactive protein levels. Estrogen has been found to enhance Survivin gene expression (86, 87), thereby inhibiting both inflammation and apoptosis, an effect that is dependent on ERα (87). Additionally, Pákó et al. (88) reported a significant decrease in Klotho protein levels in the plasma of OSAHS patients, which was also associated with nocturnal hypoxia. Nonetheless, there is insufficient direct evidence to elucidate the relationship between Klotho protein and markers of inflammation and apoptosis in OSAHS patients. Research has demonstrated that estrogen exerts varying effects on Klotho protein expression. For instance, Oz et al. (89) observed that Klotho protein levels were markedly elevated in aromatase-deficient mice, while estrogen pretreatment normalized these levels, suggesting that estrogen may lead to a reduction in Klotho protein. Conversely, Sárvári et al. (90) reported a significant upregulation of Klotho mRNA in the hippocampi of OVX rats subjected to continuous E2 treatment for 29 days. The underlying reasons for these discrepancies in findings are currently unclear, indicating the potential involvement of additional mechanisms that may influence the effects of estrogen on Klotho proteins. In summary, the regulation of apoptosis appears to be dysregulated in OSAHS and is modulated by estrogen.

The regulation of cell viability through the apoptotic process is a critical function of mitochondria. Increasing mitochondrial cytochrome c levels enhances electron flow within the respiratory chain, which may reduce the rate of ROS production (59). The B cell lymphoma-2 (Bcl-2) protein, known for its anti-apoptotic properties, plays a significant role in the mitochondria-mediated apoptotic pathway. It can diminish ROS production and mitigate apoptosis and hypoxia-induced damage by elevating cytochrome c levels within mitochondria (58). Furthermore, estrogen receptors, ERα and ERβ, regulate mitochondrial respiration and can ameliorate CIH-induced mitochondrial dysfunction and metabolic reprogramming through distinct pathways, thereby enhancing cognitive function (64, 65). Genistein, a soy isoflavone that preferentially binds to ERβ, has been shown to reduce hypoxia-induced hydrogen peroxide (H_2_O_2_) production, increase Bcl-2 expression, decrease cytochrome c release from mitochondria, and significantly lower apoptosis rates associated with hypoxia (69). This action resembles E2 while potentially mitigating the adverse effects of E2 on the reproductive system (20). Hsu et al. (91) demonstrated that resveratrol enhances the expression of Klotho mRNA and protein in mouse kidneys both *in vivo* and *in vitro*. In summary, E2 has the potential to modulate apoptosis, thereby improving the functional status of tissues and organs in patients with OSAHS. However, further research is necessary to substantiate the protective effects of E2 and to elucidate the specific molecular regulatory mechanisms involved, which could lead to the development of additional therapeutic strategies.

In contrast to apoptosis, autophagy is characterized by the self-degradation of intracellular proteins and damaged organelles, serving a crucial regulatory function during hypoxic stress. Autophagy induced by hypoxia is acknowledged as a mechanism that promotes cell survival and provides cytoprotection; however, it can also lead to cell death in specific contexts (92, 93). BNIP3, a member of the pro-apoptotic protein family, is upregulated in response to hypoxic conditions and has been implicated in the induction of autophagic cell death (93). Autophagy contributes to cell survival by dismantling dysfunctional mitochondria and decreasing ROS levels, a process contingent upon the hypoxia-induced expression of HIF-1α, which involves the HIF-1α/BNIP3 signaling pathway (94). Beclin 1 is a pivotal intersection among cellular autophagy, apoptosis, and proliferation, and it inhibits autophagy through its interaction with Bcl-2 (95). Research indicates that in genioglossus myogenic stem cells, hypoxia leads to increased HIF-1α expression and the formation of HIF-1 heterodimers, subsequently upregulating BNIP3 expression. This upregulation disrupts the Beclin 1/Bcl-2 complex, releasing Beclin 1, which then promotes autophagy under hypoxic conditions (96). Consequently, the damage to genioglossus tissue resulting from chronic hypoxia exposure in OSAHS is partially associated with autophagy-induced cell death. Numerous studies have demonstrated that E2 exerts an inhibitory effect on HIF-1α expression (17, 28, 68), aligning with the mechanisms through which OSAHS induces cellular autophagy, suggesting that E2 may modulate autophagy via the HIF-1α pathway. This hypothesis was substantiated by research conducted by Hsieh et al. (69), which revealed that E2 inhibited cardiomyocyte autophagy by suppressing hypoxia-induced HIF-1α expression, consequently limiting the expression of genes associated with autophagy. Nevertheless, a significant body of literature indicates that the regulatory effects of E2 on autophagy levels are complex and, in many instances, exhibit a stimulatory effect (97). Therefore, further investigation is warranted to elucidate the regulatory role of E2 on OSAHS-induced cellular autophagy across various tissues and organs, particularly emphasizing the mechanisms governing the interaction between E2 and HIF-1α.

### 3.4 Regulation of HIF-1α activity

HIF-1 is a heterodimeric protein consisting of HIF-1α and HIF-1β subunits, with the expression of HIF-1α being meticulously regulated by intracellular oxygen levels. Under hypoxic conditions, the stabilization of the HIF-1α protein occurs, leading to the translocation of the HIF-1 complex into the nucleus, which activates target genes crucial for cellular proliferation, survival, and differentiation (17, 62). The overexpression of HIF-1α induces a transformation of slow-twitch muscle fibers into fast-twitch muscle fibers, thereby influencing muscle tone (98). Research indicates that HIF-1α plays a significant role in regulating myogenic cell proliferation in hypoxic environments. Specifically, inhibiting HIF-1α expression in myoblasts exposed to CIH has enhanced myoblastogenesis (28). Furthermore, estrogen has been observed to downregulate HIF-1α expression in the genioglossus muscle of CIH-exposed rats, thereby improving the endurance of upper airway muscles (68). This suggests that estrogen may confer protective effects on the upper airway by inhibiting CIH-induced HIF-1α expression (17, 28, 68), with ERα mediating this effect (17). The mitogen-activated protein kinase (MAPK) signaling pathway is well-established in regulating various cellular processes, including proliferation, calcification, inflammation, and oxidative stress. A study conducted by Li et al. (17) demonstrated that the activation of the p38 MAPK pathway is further stimulated by the binding of E2 to ERα, as evidenced by a notable increase in phosphorylated p38 MAPK levels. The inhibitory effect of a p38 MAPK inhibitor on E2’s action suggests that the p38 MAPK pathway is integral to suppressing HIF-1α by E2 in myofibroblasts. Similarly, ERβ has also been shown to downregulate the hypoxia-induced increase in HIF-1α levels (65, 69).

However, research by Ding et al. (20) revealed that p38 MAPK protein levels did not significantly change in rat genioglossus muscle myoblasts subjected to hypoxia. Instead, this study found that hypoxia inhibited the expression of PI3K-Akt and ERK1/2 MAPK proteins while suppressing Bcl-2-mediated apoptosis in genioglossus myogenic cells. Notably, the effects observed with hypoxia treatment were replicated using Akt and ERK1/2 MAPK inhibitors (20), indicating that hypoxia-induced tissue damage may involve multiple pathways. Additionally, in vascular smooth muscle cells, inhibiting the MAPK pathway by E2 resulted in reduced proliferation and oxidative stress, a process associated with the upregulation of BHLHE40 (45), a transcriptional repressor, although the precise mechanism underlying this action remains unclear.

In conclusion, HIF-1α is a critical regulator for the protective effects of E2. The MAPK pathway plays a significant role in mediating the protective actions of E2 against apoptosis and oxidative stress. However, the underlying mechanisms are intricate and multifaceted, warranting further investigation to elucidate this relationship.

### 3.5 Regulation of sympathetic activity

Cyclic enhancement of sympathetic nerve activity is observed in OSAHS, as indicated by increased muscle sympathetic nerve activity (MSNA) (99), elevated urinary norepinephrine concentrations (100), and reduced heart rate variability (101), which persists during wakefulness (102). The overactivation of the sympathetic nervous system is a critical factor in the pathogenesis of cardiovascular disease among individuals with OSAHS.

Recent research has identified that the sympathetic nerve activity (SNA) associated with OSAHS is primarily linked to changes in the chemosensitivity of the carotid body (CB) induced by CIH (103, 104). The CB is the principal peripheral oxygen sensor, initiating reflex physiological responses to acute hypoxemia and facilitating ventilatory adaptation to sustained chronic hypoxemia (105). ROS produced by CIH activate chemoreflex mechanisms, thereby enhancing the chemosensitivity and responsiveness of the CB to hypoxic conditions and stimulating sympathetic nervous system activity (105, 106). This mechanism is crucial in the pathway through which CIH contributes to elevated blood pressure and arrhythmias (103, 104). Evidence suggests that pro-inflammatory mediators downstream of ROS, such as IL-1β, IL-6, and TNF-*α* (107, 108), are significant contributors to the increased sensitivity of CB chemoreceptors (105, 109). Gassmann et al. (110) demonstrated that chronic hypoxia-induced elevations in rats’ erythropoietin (EPO) levels led to the sensitization of CB chemoreceptors and enhanced hypoxic ventilatory responses. Additionally, mice with a heterozygous deficiency in hypoxia-inducible factor 1 (HIF-1 +/−) exhibited impaired carotid body function and diminished adaptive responses to chronic hypoxia (111). Consequently, the hypoxic adaptation and inflammatory responses induced by CIH may result in altered CB sensitivity, with HIF-1 potentially playing a pivotal role.

Further investigations have indicated that E2 reduces the ventilatory response to hypoxia in OVX rats without affecting responses to hypercapnia (44). E2 has been shown to inhibit the upregulation of EPO and mitigate the hypoxic ventilatory response in the CB of hypoxic rats (110). This suggests that E2 exerts a modulatory influence on the CB, thereby indirectly alleviating CIH-induced SNA. However, there is a paucity of research regarding the role of estrogen in modulating CB chemoreceptor-associated SNA, a vital aspect of the protective effects against OSAHS. The protective mechanisms of E2 against oxidative stress and inflammatory responses have been previously discussed, primarily through the downregulation of HIF levels. Future research should pursue this avenue to elucidate further the underlying mechanisms involved.

Recent research has elucidated the role of estrogen in regulating SNA in females. Notably, variations in SNA among females are linked to physiological cycles and menopause. In younger females, fluctuations in resting MSNA throughout the menstrual cycle negatively correlate with changes in plasma E2 levels (112). Premenopausal females demonstrate lower levels of sympathetic nerve activity compared to their male counterparts of the same age, while a relative increase in sympathetic nerve activity is observed post-menopause (113–115). Furthermore, many studies conducted on both animal models and human subjects have indicated that estrogen supplementation can mitigate SNA (115–119). The underlying mechanism is believed to be associated with estrogen’s influence on critical brainstem regions integral to neurocardiovascular regulation (54).

Research has identified the presence of estrogen receptors in autonomic centres of the rat brainstem, including the nucleus tractus solitarius and the rostral ventral lateral medulla (RVLM), where mRNA expression for estrogen receptor subtypes ERα and ERβ has been documented. These regions receive, integrate, and coordinate input signals to elicit appropriate autonomic responses (54, 120). For instance, localized administration of E2 into the RVLM of OVX rats has decreased sympathetic nervous tension and blood pressure (121). Additionally, E2 appears to diminish sympathetic excitation induced by the RVLM in OVX rats through the antagonism of cannabinoid receptors (118). Moreover, evidence suggests that estrogen may enhance the sensitivity of the sympathetic stress reflex, thereby inhibiting SNA (122–124). In conditions such as OSAHS, CIH typically leads to a suppression of the stress reflex, indicating that estrogen may also ameliorate SNA in OSAHS through this pathway. However, some studies have reported that E2 does not influence sympathetic stress reflex sensitivity in postmenopausal women (115, 119) This phenomenon may be related to the duration of estrogen treatment, with sympathetic activation decreasing only following chronic, rather than acute, administration. Estrogen may exert its effects by activating an inducible NO synthase signaling pathway (125). NO has been shown to inhibit noradrenergic neurotransmission at pre-sympathetic junctions and in various tissues (126, 127). Chronic estrogen treatment has been demonstrated to have a central effect in rats by enhancing the expression of neuronal NO synthase, which plays a role in the inhibitory regulation of brainstem sympathetic outflow (117, 128). Additionally, a study involving rats indicated that decreased E2 levels could diminish cardiac vagal inhibition of renal sympathetic nerves while simultaneously enhancing cardiac sympathetic excitation, leading to a significant overall increase in reflex-driven sympathetic excitability (116). This finding suggests that vagal reflexes also play a role in the estrogen-mediated regulation of sympathetic nerves.

## 4 Effect of OSAHS on estrogen levels

Numerous studies have indicated that female patients with OSAHS exhibit reduced estrogen levels (129–132), which correlate negatively with the severity of the condition (11, 133). This observation suggests a complex interplay between OSAHS and estrogen. Specifically, a decline in estrogen levels, which may result from various conditions such as polycystic ovary syndrome or premature ovarian failure, as well as from natural physiological changes like menopause, appears to facilitate the onset and progression of OSAHS. Consequently, estrogen may be considered a protective factor against this syndrome. Furthermore, the intermittent hypoxia, sleep deprivation, and other pathological processes associated with OSAHS may further contribute to decreased estrogen levels. Research conducted by Stavaras et al. (132) on pre- and postmenopausal women, after controlling for BMI, revealed a correlation between the Female Sexual Function Index (FSFI) scores and the severity of OSAHS, with significantly lower estrogen levels observed in patients with severe OSAHS. This finding implies a detrimental impact of OSAHS on female sexual function, potentially linked to variations in sex hormone levels (132). While CPAP treatment has been shown to improve sexual dysfunction in females (134, 135), some studies have reported no significant changes in sex hormone levels following such treatment (136).

In male patients with OSAHS, a robust association between the severity of the condition and sexual dysfunction has been documented (137–140). Notably, a decrease in testosterone levels has been observed (137, 139, 141, 142), alongside increases in follicle-stimulating hormone (FSH), luteinizing hormone (LH), and testosterone levels after one month of CPAP treatment (139). However, certain studies have found no significant differences in sex hormone levels between male OSAHS patients and healthy controls, nor any substantial changes after three months of CPAP treatment (140). These discrepancies may be attributed to factors such as small sample sizes, variations in age and BMI, and the circadian rhythm of sex hormone secretion, which may have influenced the results due to the lack of sleep assessments in the studies.

**Figure 4:**
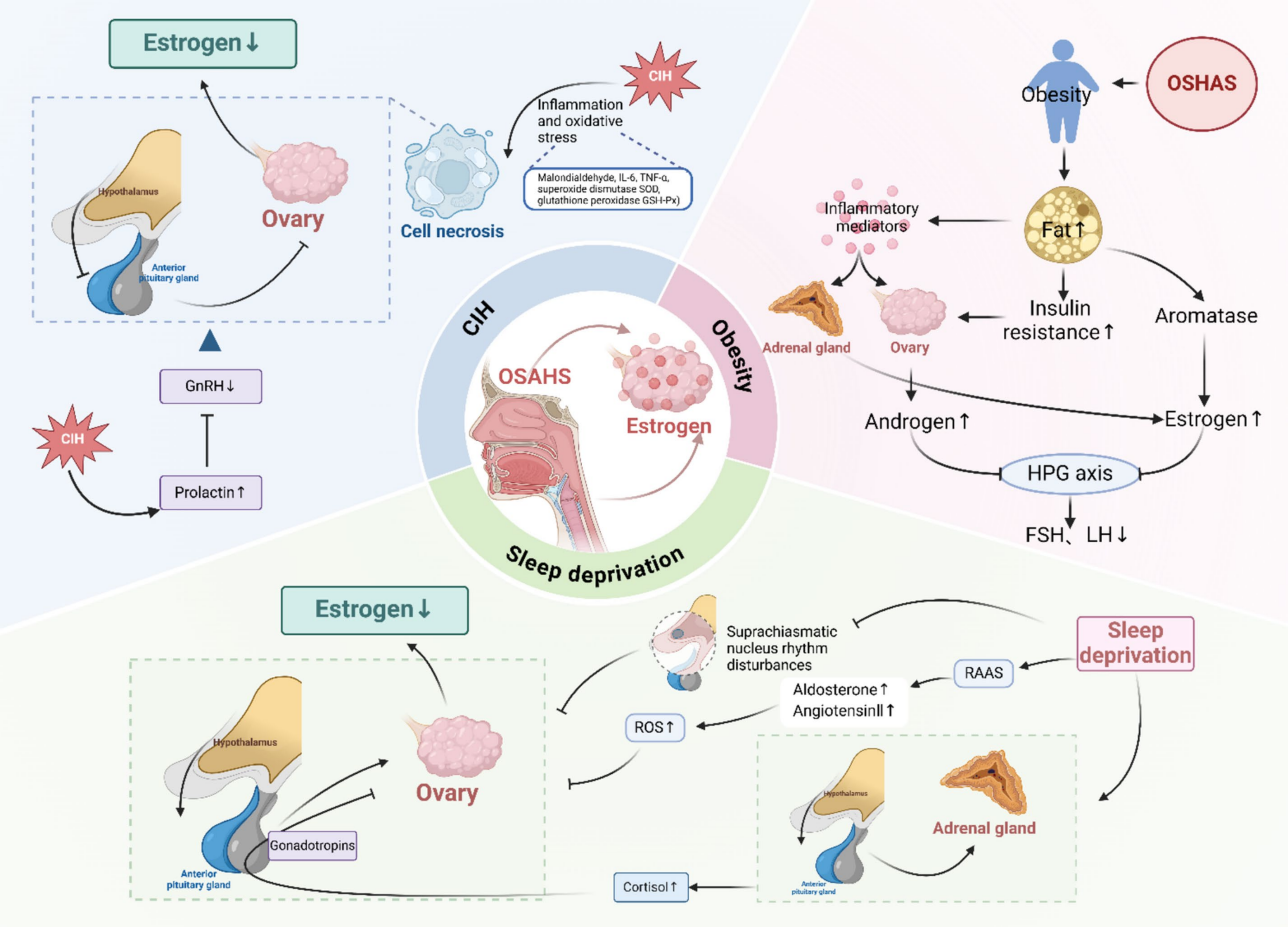
Effect of OSAHS on estrogen levels. CIH, chronic intermittent hypoxia; ROS, reactive oxygen species; GnRH, gonadotropin-releasing hormone; HPG, hypothalamic–pituitary-gonadal; FSH, follicle-stimulating hormone; LH, luteinizing hormone; RAAS, renin-angiotensin-aldosterone system. Created in BioRender.

There is a paucity of research examining the effects of OSAHS on estrogen levels in females, with the majority of studies concentrating on sexual function. It is important to note that sexual function is influenced by a multitude of factors, including psychological, neurological, endocrine, and vascular abnormalities, which cannot be solely ascribed to sex hormone levels. Therefore, further validation through rigorous clinical and animal studies is warranted. Based on the current findings, we propose the hypothesis that OSAHS may impact estrogen secretion in females and briefly discuss the underlying mechanisms involved (Figure 4).

### 4.1 CIH

CIH represents a significant pathological consequence of OSAHS, resulting in excessive oxidative stress within the body and initiating both local and systemic inflammatory responses. These responses can ultimately lead to tissue and organ dysfunction or failure (60–62). However, there is a notable paucity of research examining the impact of CIH on the structural integrity of ovarian tissue. Yang et al. (143) elucidated the adverse effects of oxidative stress and chronic inflammation on ovarian functionality, suggesting that exogenous estrogen supplementation may mitigate chronic low-grade inflammation and enhance follicular development. Furthermore, Yang et al. (144) demonstrated that CIH adversely affected the function and cellular ultrastructure of the hypothalamic–pituitary-gonadal (HPG) axis in male rats, evidenced by nuclear deformation and consolidation, vacuole formation within the cellular matrix, and mild mitochondrial swelling, which collectively resulted in diminished levels of FSH, LH, and testosterone. The study also reported elevated levels of the oxidative stress marker malondialdehyde and increased inflammatory cytokines IL-6 and TNF-*α*, alongside reduced activity of antioxidant enzymes; these alterations were ameliorated by pretreatment with reduced glutathione (144). This evidence indirectly supports the detrimental impact of CIH on the HPG axis in females.

Additional research has indicated that OSAHS is correlated with elevated prolactin levels (145), which may inhibit the secretion of hypothalamic gonadotropin-releasing hormone (GnRH), thereby indirectly influencing the release of sex hormones (146). Notably, prolactin levels can be decreased through CPAP treatment (145). These findings suggest that CIH may directly or indirectly impair the secretory function of the HPG axis via oxidative stress and inflammatory mechanisms, ultimately leading to reduced levels of sex hormones.

### 4.2 Sleep deprivation

Sleep deprivation, commonly characterized by frequent nocturnal awakenings and disrupted sleep patterns in individuals with OSAHS (1), has several implications for estrogen secretion. Firstly, estrogen secretion follows a circadian rhythm regulated by the supraoptic nucleus of the hypothalamus. Disruption of this rhythmicity due to sleep deprivation can impair the functioning of the HPG axis (15). A cross-sectional study conducted by Sowers et al. (147) involving 365 participants indicated a negative correlation between E2 levels and sleep quality in females. Furthermore, a prospective study by Michels et al. (148), which included 259 regularly menstruating women, demonstrated that an additional hour of sleep per day was associated with a significant increase of 3.9% in mean E2 concentration.

Secondly, sleep plays a crucial role in regulating the activity of the hypothalamic–pituitary–adrenal (HPA) axis. In patients with OSAHS, nocturnal hypoxic stress and sleep deprivation can activate the HPA axis, leading to disturbances in cortisol secretion rhythms (133, 142). Notably, cortisol levels have been found to correlate significantly with the AHI and minimum oxygen saturation (142), with reductions in cortisol levels observed following treatment with CPAP (149). Elevated cortisol levels can cause direct damage to ovarian tissue (150) and, when affecting the pituitary gland, can disrupt the synthesis and release of gonadotropins, thereby indirectly influencing ovarian secretory function (151).

Thirdly, sleep deprivation activates the renin-angiotensin-aldosterone system (RAAS), resulting in increased aldosterone and angiotensin II secretion (152), which may contribute to oxidative stress damage within the HPG axis. Lastly, TNF-*α* levels exhibit a circadian pattern, and sleep deprivation has been associated with elevated levels of TNF-α and IL-6 (153), which may inflict damage on ovarian tissue through the activation of inflammatory responses.

The impact of sleep deprivation on estrogen levels within the body is well-documented; however, it remains relatively underexplored in individuals diagnosed with OSAHS. The direct influence of alterations in sleep architecture on estrogen levels can be assessed through PSG monitoring.

### 4.3 Obesity

There exists a reciprocal promotional relationship between OSAHS and obesity, wherein OSAHS exacerbates endocrine dysfunction, thereby worsening obesity in individuals with obesity (1). In obese female patients, insulin resistance leads to increased ovarian production of androgens. At the same time, excess adipose tissue enhances aromatase activity, facilitating the conversion of androgens to estrogens, predominantly estrone (42, 145). Additionally, visceral adipose tissue releases significant inflammatory and immune mediators (143), stimulating the ovaries and adrenal glands to secrete these hormones (154). Elevated levels of androgens and estrogens disrupt the negative feedback mechanism of the HPG axis, resulting in the inhibition of FSH and LH secretion. Consequently, obese females may exhibit low FSH, LH, estrogen metabolites, and urinary progesterone (155). These hormonal imbalances can lead to impaired follicle development, ovulatory dysfunction, and menstrual irregularities (145, 156), often presenting as polycystic ovary syndrome (PCOS) in female patients.

A meta-analysis indicated that bariatric surgery significantly improved the signs and symptoms of PCOS and reduced free testosterone and estrogen levels in females (154). Another meta-analysis focusing on obese females without PCOS reached similar conclusions, noting that bariatric surgery ameliorated menstrual irregularities, improved insulin resistance, decreased testosterone and estrogen levels, and increased sex hormone-binding globulin levels. However, no significant changes in LH and FSH levels were observed (156). Some studies involving females (145, 157) and males (154) have reported elevated FSH and LH levels post-bariatric surgery, with this variation potentially influenced by the menstrual cycle during which the samples were collected. Sarwer et al. (157) conducted a four-year follow-up study involving 106 females post-bariatric weight loss, revealing an average weight reduction of 30% at four years post-surgery, alongside a significant decrease in estrogen levels after the procedure, with a notable increase in estrogen levels from the third year onward. This finding suggests that the inhibition of the HPG axis is alleviated following weight loss, leading to the normalization of hormone regulation. In summary, obesity results from multiple endocrine dysfunctions that inhibit the HPG axis, and weight loss surgery can mitigate pathological estrogen levels and relieve this inhibition.

In conclusion, OSAHS may influence estrogen production through various mechanisms, with sleep deprivation likely being the predominant factor. It is important to note that while examining the impact of reduced estrogen levels on OSAHS, one must also consider the reciprocal effects of OSAHS on estrogen synthesis and secretion. There is a notable scarcity of research investigating the fluctuations in sex hormone levels before and after treatment in female patients with OSAHS. This gap in the literature can be attributed to the inherent variability in the female menstrual cycle, which poses challenges in standardization and is further influenced by factors such as age, pregnancy, and the use of steroid hormones.

## 5 Exploration of the application of estrogen in the treatment of OSAHS

In the United States, more than 1.3 million women undergo menopause annually. A cross-sectional study assessed the trends in the utilization of menopausal hormone therapy among postmenopausal women in the United States from 1999 to March 2020, indicating a decrease in prevalence from 26.9% in 1999 to 4.7% in 2020 (158). During the menopausal transition, it is estimated that between 50 and 75% of women experience vasomotor symptoms, such as hot flashes and night sweats, which may lead to increased anxiety and insomnia. Additionally, over 50% of women report experiencing genitourinary symptoms (159), which can have a profound effect on their personal and social well-being (160). Estrogen therapy is recognized as the primary treatment for both vasomotor symptoms and menopausal genitourinary syndrome (159).

### 5.1 Effects of estrogen therapy on humans

The adverse effects of estrogen therapy primarily pertain to the associated risks of neoplasms, cardiovascular disease (CVD), and stroke. The existing literature on the implications of estrogen therapy for human health presents inconsistencies, particularly regarding the risks of CVD and breast cancer. Estrogen was previously advocated for the management of menopausal symptoms in women; however, its utilization significantly declined following findings from the Heart and Estrogen/Progestin Replacement Study (HERS) (161)and the Women’s Health Initiative (WHI) (162, 163) randomized trials. These studies revealed an elevated risk of CVD and breast cancer linked to the oral administration of 0.625 mg/d conjugated equine estrogens (CEE) in conjunction with 2.5 mg/d medroxyprogesterone acetate (MPA) (161–163). The concerns surrounding hormone therapy extend beyond CVD and breast cancer, as it has also been correlated with an increased risk of venous thromboembolism, overall mortality, and cancer-related deaths (164, 165). Recent research indicates that these risks may be more closely associated with progestogens, given that contraceptives containing both estrogen and progestogen, as well as progestogen-only formulations, have been shown to elevate the risk of breast cancer (166, 167). Notably, the WHI randomized trials (163, 168) and a meta-analysis (169) have demonstrated that treatment with CEE alone significantly decreases the risk of breast cancer. Furthermore, estrogen therapy does not appear to be linked to all-cause, cardiovascular, or cancer-related mortality risks (170). A meta-analysis involving over 2.5 million menopausal women suggests that oral hormone therapy is not associated with an increased risk of heart disease, and that low-dose oral and transdermal hormone therapies may confer cardioprotective benefits (171). Recent findings from the KEEPS trial indicate that oral CEE may slow the accumulation of epicardial fat tissue and impede the progression of coronary atherosclerosis (172). It is crucial to acknowledge that the studies focusing on estrogen monotherapy predominantly involve women who have undergone hysterectomy. For women with an intact uterus, the administration of CEE alone may heighten the risk of endometrial cancer, while combined treatment with MPA can mitigate this risk (173). Additionally, the WHI has reported that postmenopausal women receiving estrogen alone face an increased risk of stroke (174), as well as heightened incidence and mortality rates of ovarian cancer (173).

The etiology of organ damage linked to hormone therapy may be influenced by the age at which treatment commences, especially in relation to the age of menopause. Additionally, factors such as the type, dosage, and method of administration of estrogen, along with the concurrent use of progestogens, may also play a significant role (171).There is substantial evidence indicating that estrogen therapy may confer cardioprotective benefits when initiated around the time of menopause. In contrast, its initiation during the late menopausal period (more than ten years post-menopause) may be detrimental (171). This phenomenon can be attributed to the less favorable CVD risk profile observed in women who are further along in the menopausal transition, rendering them more susceptible to CVD (175). A secondary analysis of the WHI revealed that women aged 50 to 59 years, or those who are less than ten years post-menopause, exhibited a decreased risk of heart disease, a lower likelihood of mortality from all causes, and no significant increase in stroke risk, in contrast to women who commenced hormone therapy after the age of 60 (176). Furthermore, early initiation of estrogen replacement therapy has been shown to significantly impede the progression of coronary atherosclerosis, diminish the risk of colon cancer, and potentially reduce all-cause mortality by 20–40% (177). Conversely, the initiation of hormone replacement therapy a decade post-menopause may elevate the risk of cardiovascular disease, among other health concerns (177). Consequently, the timing of hormone therapy initiation relative to menopause is a critical determinant of its effects on chronic disease risk (165). The duration of estrogen’s absence prior to therapy is particularly relevant, as it influences the risk of atherosclerosis. Additionally, it is essential to note that while estrogen is not inherently carcinogenic, it can promote the growth of pre-existing tumors; thus, timely screening for tumors is essential for menopausal women, particularly prior to the commencement of hormone therapy (165). Unless contraindicated, patients may opt to continue hormone therapy until the associated risks surpass the benefits (165). Regular reassessment of a woman’s health status is necessary throughout treatment.

In summary, the formulation of a personalized approach to estrogen therapy is of paramount importance (178). A comprehensive evaluation of various factors, including the dosage of the medication, the administration route, the specific type of hormonal agent utilized (whether combined estrogen or progestin), the timing of treatment initiation, the patient’s age, her cardiovascular disease history, and the thromboembolic characteristics associated with estrogen and progestin, is essential for postmenopausal hormone replacement therapy (171). The debate surrounding the advantages and disadvantages of estrogen therapy remains unresolved.

### 5.2 Clinical studies of estrogen therapy for OSAHS

Wesström et al. (179) administered a regimen of hormone replacement therapy consisting of 2 mg/d of estradiol and 0.5 mg/d of trimegeston orally for a duration of 5–6 weeks to four postmenopausal women and one perimenopausal woman diagnosed with OSAHS via PSG monitoring. The results indicated a significant average reduction of 75% in the AHI post-treatment. In a separate study conducted by Keefe et al. (180), treatment with oral estrogen (2 mg/d) and combined progesterone (10 mg/d) yielded reductions in AHI of 25 and 50%, respectively. Additionally, Heinzer et al. (8) reported that postmenopausal women undergoing hormone replacement therapy exhibited comparatively lower AHI values. Conversely, Cistulli et al. (181) conducted a study involving 15 postmenopausal women with moderate OSAHS, administering estrogen (either oral CEE at 0.625 mg/d, estradiol valerate at 1 mg/d, or transdermal estradiol at 8 mg/week) in conjunction with combined progesterone (MPA at 2.5–10 mg/d) over a period of 50 days, and found no significant reduction in sleep-disordered breathing events. Notably, both the studies by Keefe and Cistulli were unblinded and lacked a control group. Additionally, Wesström’s investigation was a small-sample clinical trial without a placebo control, leading to ongoing debates regarding the validity of the results. Currently, there is insufficient conclusive evidence to ascertain the efficacy of estrogen alone or estrogen-progestin combination therapy in postmenopausal women with OSAHS. Nevertheless, findings from various animal studies and clinical investigations suggest that estrogen therapy may confer a protective effect against the onset and progression of OSAHS and its associated complications in postmenopausal women, with the combination of progestin potentially enhancing therapeutic efficacy (6). Further extensive and methodologically rigorous clinical studies are warranted to substantiate these conclusions.

### 5.3 Basic research on estrogen therapy for OSAHS

The impact of CIH on female patients with OSAHS is influenced by various stages of ovarian hormone production, including the physiological menstrual cycle, pregnancy, and menopause. This observation indicates that hormonal therapies aimed at contraception, alleviating menopausal symptoms, or treating estrogen receptor-positive breast cancer may interact with CIH, potentially modifying the responses of other tissues such as the lungs, cardiovascular system, brain, and kidneys in female OSAHS patients (61). Research has demonstrated that low doses of E2 can reduce inflammatory and immune responses (67), while physiologically elevated levels of E2 may intensify these responses (182). In a study conducted by Huang et al. (13), the subcutaneous implantation of E2 silicone capsules demonstrated that supplementation with low concentrations of E2 (30 mg/mL) significantly diminished the apnea reflex induced by CIH in OVX rats, as well as the afferent response to chemical stimuli and the pulmonary inflammatory response; conversely, higher concentrations of E2 (50 and 150 mg/mL) did not yield significant effects.

These findings suggest that the influence of estrogen on the inflammatory response induced by CIH may be concentration-dependent, warranting further investigation to determine whether similar concentration-dependent effects of estrogen are observed in other tissues.

Recent investigations into the utilization of phytoestrogens have yielded promising results. Genistein, a polyphenolic nonsteroidal compound derived from plants, is among the most extensively studied phytoestrogens, exhibiting estrogen-like bioactivity without significant toxic effects during prolonged use (183). A study conducted by Zhou et al. (28) demonstrated that genistein enhances the endurance of upper airway muscles and mitigates airway collapse by down-regulating the expression of HIF-1α in the genioglossus of CIH rats. However, this effect was less pronounced than that of estrogen. Subsequent research indicated that genistein reduces oxidative stress and myogenic apoptosis through various mechanisms, including the regulation of ROS, lipid peroxidation, Bcl-2, and the apoptosis marker caspase-3 via the PI3K-Akt and ERK1/2 MAPK signaling pathways, with its effects remaining unaffected by estrogen receptor antagonists [28]. This suggests that the protective role of genistein against hypoxia-induced damage to genioglossus myogenic cells may operate through a non-genomic pathway rather than a genomic pathway mediated by estrogen receptors (28).

Moreover, genistein exhibits a high affinity for ERβ, while ERα is predominantly distributed in skeletal muscle. Experimental findings indicate that low concentrations of genistein confer a more pronounced protective effect against adult myocyte injury, suggesting that this protective effect may be both tissue-specific and concentration-dependent (20). Further research is warranted to elucidate its physiological effects and mechanisms in other tissues. Additionally, other phytoestrogens, such as resveratrol dimer (a derivative of resveratrol), which possesses superior estrogenic properties and minimal cytotoxicity, can enhance ERα expression by binding to ERα, thereby activating the p38 MAPK pathway to inhibit HIF-1α expression, ultimately improving the function of the genioglossus (17).

In conclusion, the utilization of estrogen for the management of OSAHS presents specific practical considerations; however, there is a deficiency of robust clinical evidence to substantiate its efficacy. Phytoestrogens exhibit various protective effects against OSAHS and may mitigate the adverse effects of conventional estrogens. Further investigation into their mechanisms of action is essential, as this research holds considerable importance for preventing and treating OSAHS in postmenopausal women. Additionally, hormone replacement therapy for postmenopausal women with OSAHS carries inherent risks and potential benefits.

## 6 Conclusion

A substantial body of evidence indicates that the chronic systemic inflammatory response associated with OSAHS impacts the body’s estrogen levels. Notably, estrogen serves a crucial protective function in the context of OSAHS. Disruption of this balance may exacerbate the condition. Future research should focus on a more comprehensive examination of the interplay between OSAHS and estrogen. The advancement and utilization of phytoestrogens have partially mitigated the severe complications associated with traditional estrogen therapy, positioning them as a promising therapeutic adjunct for postmenopausal patients with OSAHS. Given the significant relationship between OSAHS and estrogen, we propose that a combination therapy involving CPAP and E2 could be explored to substantially enhance the clinical symptoms of affected individuals, at least in the short term. However, this approach necessitates robust empirical support through extensive experimental data.
